# Assessing the Role of Transvaginal Sonography in Post-menopausal Bleeding: A Less Invasive Approach to Identify Endometrial Carcinoma

**DOI:** 10.7759/cureus.65608

**Published:** 2024-07-28

**Authors:** Indrakshi Saha, Sachin Wankhede, Sarika Thakare, Gaurang Narayan, Akash A Sawant, Ayushi Gupta, Akash G Ingle, Sana U Rajkotwala

**Affiliations:** 1 Obstetrics and Gynaecology, Indira Gandhi Government Medical College and Hospital, Nagpur, Nagpur, IND; 2 Obstetrics and Gynaecology, Government Medical College and Hospital, Nagpur, Nagpur, IND

**Keywords:** post-menopausal bleeding, endometrial hyperplasia, endometrial thickness (et), transvaginal ultrasound scan, endometrial carcinoma

## Abstract

Background

Post-menopausal bleeding (PMB) is a very common complaint seen in current practice. Endometrial carcinoma (EC) commonly presents with PMB. Endometrial biopsy is the tool for definitive diagnosis, but it is invasive. Transvaginal sonography (TVS) is a non-invasive tool that can help us in the initial evaluation of such patients.

Methods

A prospective observational study was conducted on 76 women with PMB. TVS and histopathological study, along with basic evaluation and investigations, were performed on all participants, followed by necessary treatment and follow-up. Data collected were studied and statistically analyzed.

Results

A maximum of 27.63% (n=21) of patients had endometrial atrophy causing their PMB. Proliferative endometrium was observed in 21.06% (n=16) of cases, 13.15% (n=10) of women had secretory endometrium, 23.68% (n=18) had simple endometrial hyperplasia, 3.94% (n=3) had complex endometrial hyperplasia without atypia, and another 3.94% (n=3) had complex endometrial hyperplasia with atypia. Further classifying, women with benign hyperplasia included 27.63% (n=21) and those with atypical hyperplasia included 3.94% (n=3). Out of the 5.26% (n=4) patients diagnosed with EC on histopathology, TVS identified carcinoma in 75% (n=3) cases. This indicates that the sensitivity and specificity of TVS in detecting EC are 75% and 100%, respectively. The positive predictive value (PPV) is 100%, the negative predictive value (NPV) is 98.63%, and the accuracy is 98.68%.

Conclusion

If the cut-off for endometrial thickness is set at 4 mm, then TVS proves to be an effective and reliable tool for screening and diagnosing EC. It can thus serve as a safe threshold to screen patients with PMB using TVS.

## Introduction

Post-menopausal bleeding (PMB) accounts for 5% of all gynecological outpatient visits [1,2]. Over the past few decades, with an increasingly aging population, the number of post-menopausal women has significantly risen, making it a critical concern in current gynecological practice. Unless proven otherwise, it is often attributed to endometrial carcinoma (EC) [3]. While endometrial biopsy remains the gold standard for diagnosis, it is invasive [4]. This study aims to determine whether transvaginal sonography (TVS) can effectively serve as a less invasive diagnostic tool to identify which women with PMB should undergo endometrial biopsy and which can be managed with follow-up.

## Materials and methods

The study was conducted over a period of two years, from October 2020 to October 2022, in a tertiary care hospital under the Department of Obstetrics and Gynaecology. This was a prospective observational study involving 76 women who presented with PMB. All females presenting with PMB and seeking medical care at the tertiary health center were included in the study after obtaining their verbal informed consent.

The study aimed to include all eligible participants within the study period. The estimated sample size for the study was calculated using the formula for proportions, taking into account the expected prevalence of PMB, the desired confidence level, and the margin of error. Given a prevalence of 1.8% (0.018) [5], a confidence level of 95% (Z = 1.96), and a margin of error of 5% (0.05), the sample size was determined using the formula $n= \left [ Z^{2} \times p\times \ \left ( 1-p \right ) \ \right ] \div E^{2}$. Substituting the values, the estimated sample size was approximately 28. However, the study included 76 participants to ensure robustness and account for potential dropouts or non-responses.

The study excluded patients who were on hormone replacement therapy, on tamoxifen for breast cancer, patients suffering from liver disorders and coagulation disorders, patients with neglected intrauterine devices (IUDs) or pessary, and patients with established local causes of bleeding (e.g., vulval/vaginal causes).

Data was collected using a semi-structured questionnaire, which included basic demographic details such as age, marital status, education, and occupation. Participants were also asked about risk factors and symptoms related to PMB, including onset and age of occurrence. Written informed consent was obtained from all participants before the interview, and utmost care was taken to maintain their privacy and confidentiality throughout the study. Anthropometric records were additionally taken. All the patients were subjected to a screening trans-vaginal ultrasound examination by the radiologist. Samples of their endometrium were collected either on a daycare basis by Pipelle biopsy, abiding by the standard operating procedures of the hospital, or during therapeutic dilatation and curettage. In these cases, samples from the procedure were collected and sent for histopathological examination.

Data was entered in MS Excel software version 20 (Microsoft® Corp., Redmond, WA, USA) and analyzed using Statistical Package for the Social Sciences (IBM SPSS Statistics for Windows, IBM Corp., Version 18.0, Armonk, NY, USA). Categorial variables were expressed as means and standard deviations. Data was represented in figures, tables, and pie charts.

## Results

A total of 76 post-menopausal women who presented with bleeding were studied. The mean age of the study subjects was 57.43 years, the mean parity was 3.17, and the mean BMI was 26.00. The mean age at menopause was 48.85 years, and the mean duration after menopause before developing PMB was 8.58 years. Among the patients with PMB, 26.31% (n=20) were hypertensive, 5.26% (n=4) were diabetic, 8% (n=6) were obese, and 44% (n=33) were free of any comorbidities. The mean endometrial thickness (ET) in these 76 patients was 8.026 mm. Additionally, 65.78% (n=50) of the patients had an ET of more than 4 mm, while 34.21% (n=26) had an ET of less than 4 mm. Biopsy results showed that 5.26% (n=4) of the patients had EC as the cause of their PMB.

**Figure 1:**
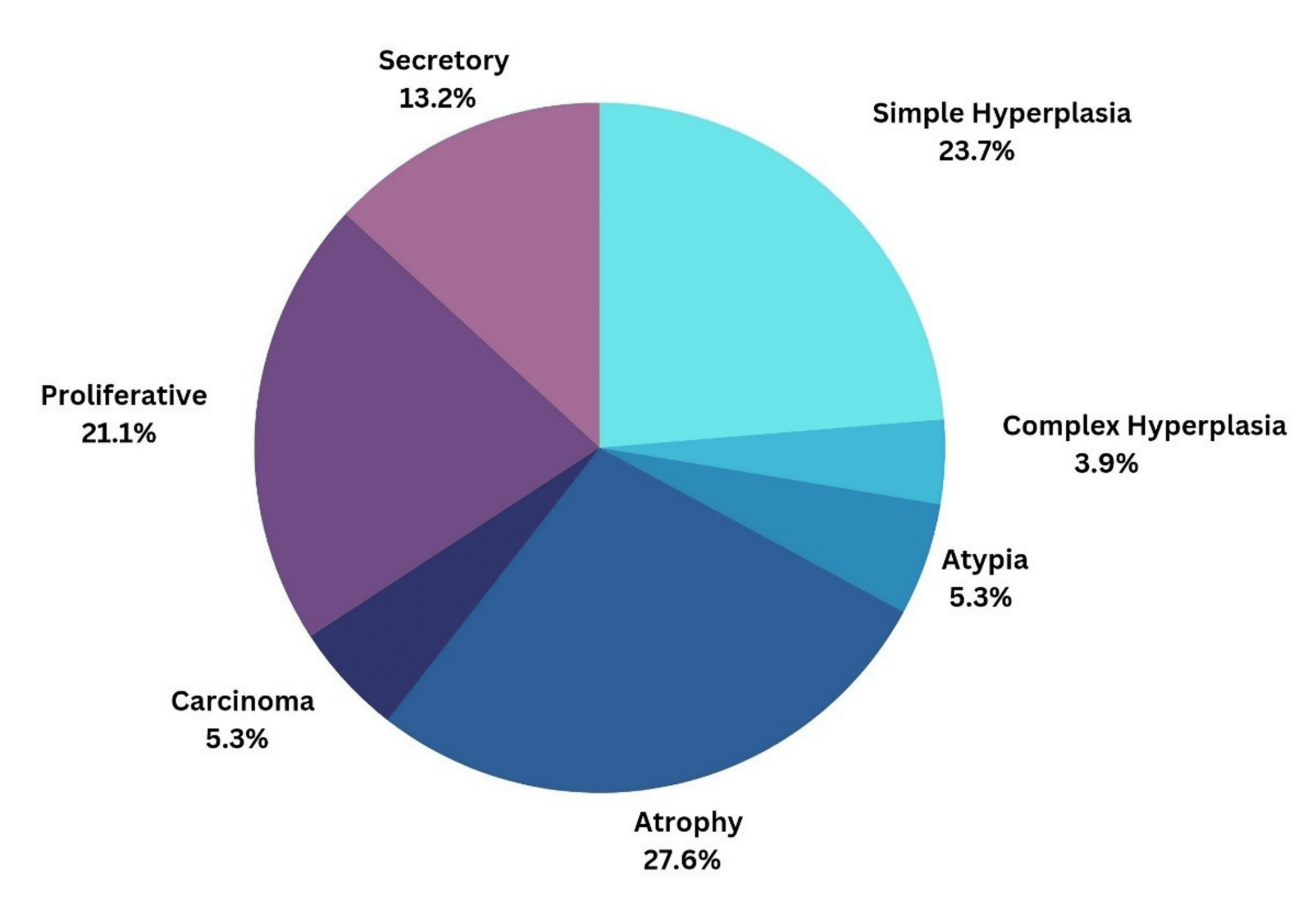
Pie Chart Showing the Causes of Post-menopausal Bleeding in the Study

Figure 1 is a pie chart showing common causes of PMB in the study. It was observed that the most common cause of PMB was endometrial atrophy, affecting 21 patients (27.63%). Other histopathology findings included proliferative endometrium in 21.06% (n=16), secretory endometrium in 13.15% (n=10), simple endometrial hyperplasia in 23.68% (n=18), complex endometrial hyperplasia without atypia in 3.94% (n=3), and complex endometrial hyperplasia with atypia in 5.34% (n=4). Further classifying, women with benign hyperplasia included 27.63% (n=21) and those with atypical hyperplasia or endometrial intraepithelial neoplasia (EIN) included 3.94% (n=3).

TVS revealed a normal thin endometrium in 57.89% (n=44) of patients with PMB, a polyp in 3.94% (n=3) of patients, fibroids in 11.84% (n=9) of patients, and adenomyosis in another 3.94% (n=3) of patients. A thick regular endometrial pattern was suggested in 30.26% (n=23) of patients, while a thick irregular endometrium was seen in 3.94% (n=3) of patients.

Out of the four patients diagnosed with EC on histopathology, 75% (n=3) were correctly identified by TVS as having carcinoma. The remaining patient was only indicated to have hyperplasia on TVS. This results in a sensitivity of 75% and a specificity of 100% for TVS in detecting EC. The positive predictive value (PPV) was 100%, and the negative predictive value (NPV) was 98.63%.

**Figure 2:**
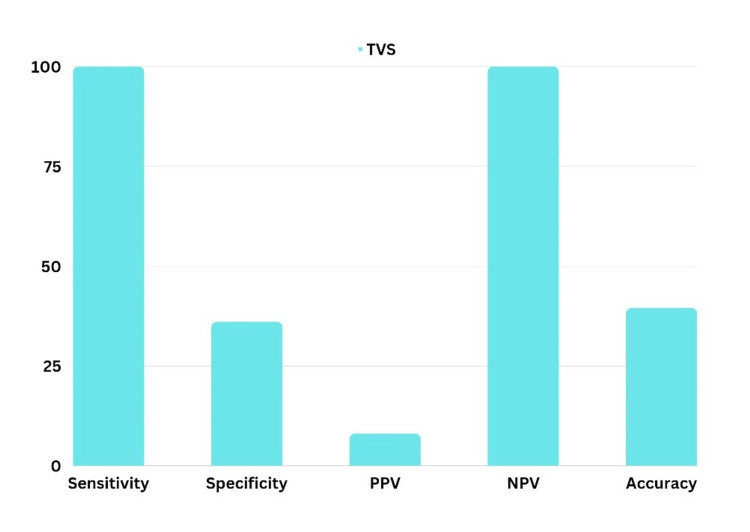
Bar Diagram Showing the Sensitivity, Specificity, PPV, NPV, and Accuracy of Transvaginal Sonography as a Screening Tool for Endometrial Carcinoma PPV: positive predictive value; NPV: negative predictive value; TVS: transvaginal sonography

Figure 2 suggests that when the ET threshold was taken as 4 mm, the specificity of TVS in screening EC was 36% but the sensitivity was 100%. The PPV of EC was 8% only but the NPV was 100% bringing accuracy to 39.47% with an ET threshold of 4 mm.

## Discussion

PMB is a prevalent clinical issue affecting approximately 3% of post-menopausal women. This study evaluates PMB, focusing on EC and associated risk factors. PMB can be caused by both benign and malignant conditions, making the differentiation crucial to avoid misdiagnosis and ensure timely treatment.

The incidence of PMB decreases with increasing age [3], a trend confirmed by the present study. The highest number of cases (30, or 39.47%) were in the age group of 51 to 60 years, while only seven cases (9.21%) were observed in women above 70 years of age. Additionally, a significant proportion of patients (32 out of 76, or 42.10%) experienced bleeding within five years of menopause.

Obesity is a crucial risk factor for EC due to the increased peripheral conversion of androgens to estrogen by body fat, leading to more unopposed estrogen and abnormal endometrial proliferation. In this study, 10.52% of patients were obese (BMI > 30 kg/m²), which contrasts with the findings of Nirupama et al. [6], where 45% of their cases were obese, and Viswanathan et al. [7], who found obesity in 6.6% of cases. The mean BMI in this study was 26.001, aligning closely with other research findings. Additionally, 5.26% of patients had diabetes, 26.51% had hypertension, and 9.21% had both. These conditions are recognized risk factors for EC due to their association with increased estrogen levels [7,8].

The average age of menopause in the study was 48.85 years. PMB typically began 8.58 years post-menopause, with a range of 1 to 22 years. This data aligns with other studies, highlighting the critical period shortly after menopause as a window for increased vigilance. The mean age for EC diagnosis in the study was 57.43 years, reflecting an increased risk with advancing age. Nulliparity is a known risk factor for EC due to prolonged exposure to unopposed estrogen. This study had a mean parity of 3.17, consistent with other studies. Notably, none of the EC cases in the current study were nulliparous. The study's findings on BMI, comorbidities, age at menopause, and mean duration of menopause were compared with other research, showing consistency in the risk factors and demographic characteristics associated with EC [9,10].

TVS is highly sensitive but less specific for detecting abnormal endometrial conditions. ET greater than 4 mm is a significant marker for EC, with a sensitivity of 100% and specificity of 36% when using this threshold. The NPV is 100%, indicating that patients with ET below 4 mm are unlikely to have EC. TVS was effective in detecting atrophic endometrium and endometrial hyperplasia but showed limitations in identifying EC compared to histopathology. The correlation was strong for benign conditions but less reliable for malignancies. TVS also identified myometrial lesions such as leiomyoma and adenomyosis, with histopathology confirming most diagnoses. The accuracy of TVS in detecting these conditions was comparable to other studies [11,12].

Among the patients with EC, the majority (three out of four, or 75%) had an ET greater than 12 mm. No endometrial cancer was found in 34.21% of patients with an ET of less than 4 mm. This study supports earlier reports that endometrial cancer is uncommon in women with a thin endometrium (less than 4 mm) as measured by TVS.

The most common benign cause of PMB is endometrial hyperplasia. In this study, simple endometrial hyperplasia was found in 23.68% of cases. Endometrial hyperplasia, an estrogen-dependent condition, shares risk factors with EC. Simple endometrial hyperplasia has only a 1% risk of progressing to EC. Atypical hyperplasia or EIN was found in 5.26% of cases, which has a 25-30% chance of progressing to invasive carcinoma. Women with simple hyperplasia typically respond to hormonal treatment, while those with atypical hyperplasia should consider a total hysterectomy [13].

In the present study, histopathology revealed four cases of EC. The ET in these patients was 15 mm, 18 mm, and 8 mm. TVS indicated EC in three of these four patients, showing irregularly thickened endometrium and increased vascularity on color Doppler, with one patient also exhibiting loss of the endo-myometrial junction.

This study found that using a 4 mm cut-off for ET minimizes the risk of missing EC, suggesting it as a safe threshold for screening patients with PMB. Kaul et al. [14] used a 5 mm cut-off in their study, finding that the sensitivity and specificity of TVS were 80% and 100%, respectively, with a PPV of 76.9% and an NPV of 100%. According to Sedeq et al. [15], among 107 patients with PMB and an ET of less than 4 mm, only one was diagnosed with EC on follow-up, representing 0.9% (1 in 107). In contrast, 20.1% of women with an ET greater than 4 mm had EC.

The study underscores the importance of using TVS as a primary screening tool for PMB, given its high sensitivity. However, its specificity limitations necessitate follow-up histopathological confirmation, especially for detecting malignancies. Recognizing risk factors such as obesity, comorbidities, age, and parity is crucial for early diagnosis and management of EC. The study aligns with existing literature, reinforcing the need for comprehensive clinical evaluation in post-menopausal women presenting with bleeding.

The strength of the study is the geographic location as it would help generate local data and the limitations of the study include its smaller sample size and loss to the follow-up component of such patients after establishing the diagnosis and the treatment.

## Conclusions

TVS is an excellent primary investigation for PMB due to its ease of use, affordability, and non-invasive nature. It effectively measures ET without any side effects. When using a cut-off of 4 mm for ET, TVS proves to be a reliable and efficient tool for screening and diagnosing EC. This threshold allows for a conservative approach in women with ET less than 4 mm, reducing the need for invasive procedures like curettage. For cases where ET exceeds 4 mm, an endometrial biopsy and histopathological correlation with TVS findings are necessary. Women showing endometrial thickening along with other positive ultrasound findings, such as increased vascularity on color Doppler, altered endometrial homogeneity, particulate fluid, or a significantly thickened endometrium, should undergo further investigation via endometrial biopsy. Thus, measuring ET through TVS is a reliable method for excluding malignancy, demonstrating a high correlation with histopathological results.
